# Spatial–Semantic and Temporal Attention Mechanism-Based Online Multi-Object Tracking

**DOI:** 10.3390/s20061653

**Published:** 2020-03-16

**Authors:** Fanjie Meng, Xinqing Wang, Dong Wang, Faming Shao, Lei Fu

**Affiliations:** 1 Department of Mechanical Engineering, College of Field Engineering, Army Engineering University of PLA, Nanjing 210007, China; beilimeng1992@163.com (F.M.); dyhkxywangdong@163.com (D.W.); shaofaming@163.com (F.S.); 2 Department of Armament Science and Technology, College of Field Engineering, Army Engineering University of PLA, Nanjing 210007, China; fulei10@mails.jlu.edu.cn

**Keywords:** deep learning, video processing, spatial-temporal attention, multi-object tracking, autonomous vehicle

## Abstract

Multi-object tracking (MOT) plays a crucial role in various platforms. Occlusion and insertion among targets, complex backgrounds and higher real-time requirements increase the difficulty of MOT problems. Most state-of-the-art MOT approaches adopt the tracking-by-detection strategy, which relies on compute-intensive sliding windows or anchoring schemes to detect matching targets or candidates in each frame. In this work, we introduce a more efficient and effective spatial–temporal attention scheme to track multiple objects in various scenarios. Using a semantic-feature-based spatial attention mechanism and a novel Motion Model, we address the insertion and location of candidates. Some online-learned target-specific convolutional neural networks (CNNs) were used to estimate target occlusion and classify by adapting the appearance model. A temporal attention mechanism was adopted to update the online module by balancing current and history frames. Extensive experiments were performed on Karlsruhe Institute of Technologyand Toyota Technological Institute (KITTI) benchmarks and an Armored Target Tracking Dataset (ATTD) built for ground-armored targets. Experimental results show that the proposed method achieved outstanding tracking performance and met the actual application requirements.

## 1. Introduction

Multi-object tracking (MOT) is one of the most fundamental capabilities of unmanned aerial vehicles (UAV), armored scout cars (ARSVs) and other platforms [1,2]. Among them, MOT based on digital image sensing has become a hotspot for research, as it would allow dynamic environments to be captured through an accurate tracking the movement of multiple targets object. Most existing multi-object tracking approaches adopt a two-step procedure. In the first step, the potential candidates are located using a detection algorithm. In the next step, the potential candidates are estimated and linked across different frames. The challenges of multi-object tracking can be summarized as following:

- A tracking system is required to deal with occlusion and insertion. The digital image sensor has a limited receptive field, which means that occlusion and insertion are common. In a receptive field, targets entering and leaving result in boundary insertions, and the close spatial positions of targets in the field result in the occlusion of targets.
- The ability to track small targets is highly important. Small targets are very common in real-life situations, and the ability to recognize small targets gives tracking system a longer response time. This is quite a significant challenge for conventional tracking-by-detection strategies.
- The tracking system is required to be robust. Some scenes, including jungle, desert, and grassland, are more complicated than general scenarios. Dust caused by movement adds complexity to a background.
- There is no ready-made MOT dataset available for armored targets. Compared with general multi-object tracking, the tracking of multi-armored targets is more challenging after adopting camouflage or smoke shielding to avoid exposure.

**Figure 1:**
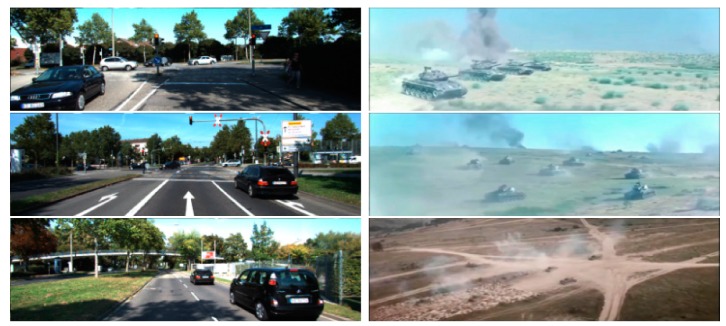
Typical frames including vehicles in KITTI tracking benchmarks and armored targets from our Armored Target Tracking Dataset (ATTD).

Figure 1 presents typical frames including vehicles in KITTI tracking benchmarks and armored targets from our Armored Target Tracking Dataset (ATTD).

In consideration of the challenges mentioned above, we proposed an online multi-object tracking method based on a spatial–temporal attention mechanism (STAM) [3]. In order to reduce computation, we proposed an Offline Candidates Recommendation Module that was based on a novel spatial-attention map, leveraging semantic features to determine suspect areas as opposed to the sliding windows and dense anchoring scheme in STAM. This strategy can filter out 80% of the invalid areas while maintaining the same recall rate. Considering the irregular movement of armor targets, a novel Motion Model was proposed to analyze the motion trajectories of history frames and predict the precise current position of target. Online-trained target-specific convolutional neural networks (CNNs) were used to estimate the classification and occlusion for each candidate in the same manner as STAM. In order to balance the effects of current and history frames during online training, a temporal attention mechanism was introduced to update the parameters of the target-specific CNNs. Finally, aiming to establish a ready-made MOT dataset for armored targets, we built an Armored Target Tracking Dataset (ATTD) via actual data collection and network downloading. Several experiments were conducted to verify the proposed method on the vehicle-target dataset KITTI and the armored-target dataset ATTD.

Our main contributions are summarized as follows. Firstly, an Offline Candidates Recommendation Module based on a spatial attention mechanism was proposed that could produce fewer false negatives and greatly reduce the computation. Secondly, a novel Motion Model was proposed to locate which candidates provide a full consideration to the possible motion of the target and fit more complex movements. Thirdly, an Armored Target Tracking Dataset (ATTD) was built to address the lack of a ready-made MOT dataset for armored targets.

The rest of our paper is organized as follows: In Section 2, we introduce the related work. In Section 3, we provide an overview of the method then present the details of our multi-object tracking method. The experimental evaluation is provided in Section 4. Finally, we present some conclusions and suggest future work in Section 5.

## 2. Related Work

### 2.1. Single-Object Tracking

Tracking is a fundamental task in any video processing that requires some degree of reasoning about objects of interest [4,5,6]. The methods of object tracking can be divided into two categories: the method suitable for single-object tracking [7,8,9], and the method suitable for multi-object tracking [10,11,12]. Until very recently, the most popular single-object tracking method trained a discriminative classifier online (then updated online) using ground-truth information from the first frame to achieve target tracking. The appearance of a target is often the only link to a video frame. These discriminative classifiers usually have a filter- or deep-neural-network-based structure. A few years ago, Bolme et al. [13] proposed Correlation Filtering, a simple algorithm that permits discrimination between the template of an arbitrary target and its 2D translations, to quickly distinguish a single object from the background. Correlation Filtering and its improved tracking method [14,15,16,17] are widely used in various tracking applications. However, the Correlation Filtering method has poor performance in tracking targets with obvious deformations. Recently, with the great success of deep convolutional neural networks (CNNs) [18,19,20,21,22], a discriminative offline classifier represented by the Siamese [9] model has been widely applied in cases of single-object tracking. During testing, the Siamese model formulates tracking as a convolutional feature cross-correlating between a target template and a search region. Wang et al. [8] improved the offline training procedure of the popular fully convolutional Siamese approach for single-object tracking by augmenting their loss with a binary segmentation task. Li et al. [7] used comprehensive theoretical analysis and experimental validations to break the Siamese tracker’s restriction against deep networks and take advantage of features. They integrated deep networks into the Siamese network to make the network more robust. In conclusion, single-object tracking focuses on tracking the contours of a target to determining its center position.

### 2.2. Multi-Object Tracking

As opposed to single-object tracking, the core topics being researched for multi-object tracking are the occlusion of multiple targets, the insertion of new targets around boundaries and the disappearances of targets from a scene [23,24,25]. The appearance cues of obscured targets used for training are polluted when the spatial positions of targets are too close together in a scene. In these cases, a single-object tracker will update the appearance model with the corrupted samples and gradually drift to the occluder [3]. Furthermore, single-object trackers cannot deal with a new target being inserted in the receptive field, and a new ground-truth needs to be added, which is difficult to achieve. At present, most state-of-the-art MOT methods adopt the strategy of tracking-by-detection [11,26,27,28], which is a two-step procedure composed of a detection module and a tracking module. In the detection module, candidates are recommended in each frame. Then, the candidates are estimated in tracking module. In Son et al. [29], a quadruplet convolutional neural network is proposed for multi-object tracking that can learn to associate object detections across frames using quadruplet losses. Dawei et al. [11] proposed a multi-scale object detector to augment the Single-Shot multi-box Detector (SSD) with temporal regions of interests (ROIs). However, the spatial–temporal relationship of targets is not involved in their method. Chu Q et al. [3] used a spatial–temporal attention mechanism to track multiple objects. They built a Motion Model based on the correlation between current and history frames to recommended candidates. The spatial attention mechanism is used to estimate the occlusion and the temporal attention mechanism is used to realize the online update of the tracking module.

The insertion of new targets around boundaries is disregarded in this work, and the advantages of deep convolutional neural networks are not applied. In addition, a linear Motion Model cannot be applied to a complicated motion.

## 3. Proposed Method

### 3.1. Overview of Our Method

**Figure 2:**
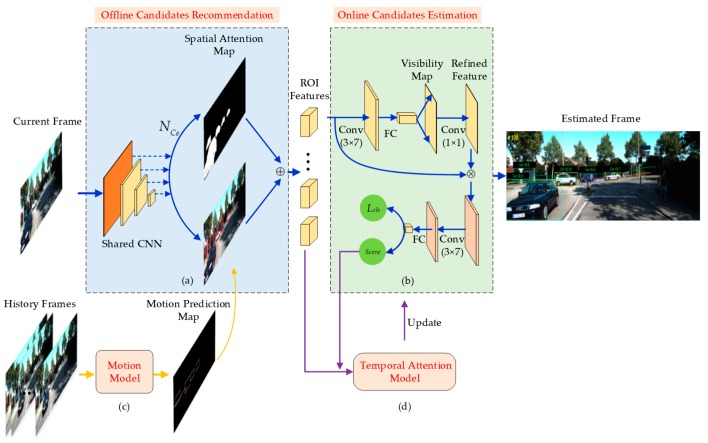
Overview of our method. (**a**) Offline Candidates Recommendation Module; (**b**) Online Candidates Estimated Module; (**c**) Motion Model; (**d**) Temporal Attention Model. ⨁ demotes combination.

With a focus on multi-object tracking, we proposed a multi-object tracking method based on the spatial–temporal attention mechanism shown in Figure 2. The MOT method consists of four parts: (a) an Offline Candidates Recommendation Module; (b) an Online Candidates Estimation Module; (c) a Motion Model; and (d) Temporal Attention Model. Firstly, the current frame is sent into the Offline Candidates Recommendation Module, which is trained offline to predict the suspect areas of candidates. Replacing the sliding window or dense anchoring scheme, we use a spatial-attention map to filter out most areas (such as sky and grass) that are irrelevant to the targets of interest in the offline module. Meanwhile, the insertion of new target around the boundary is solved. Next, a novel Motion Model analyzes the motion curve of the target in the history frames and assists the offline module to determine the location and shape of the bounding box. The ROI features of the candidates are determined and sent to the Online Candidates Estimation Module. In the online module, estimation is operated for each ROI feature, including classification and occlusion. The Temporal Attention Model updates the Online Candidates Estimation Module by balancing the positive and negative samples of the history and current frames. Finally, the multi-object position and classification results of the current frame are evaluated and the model is updated.

### 3.2. Offline Candidates Recommendation Module

**Figure 3:**
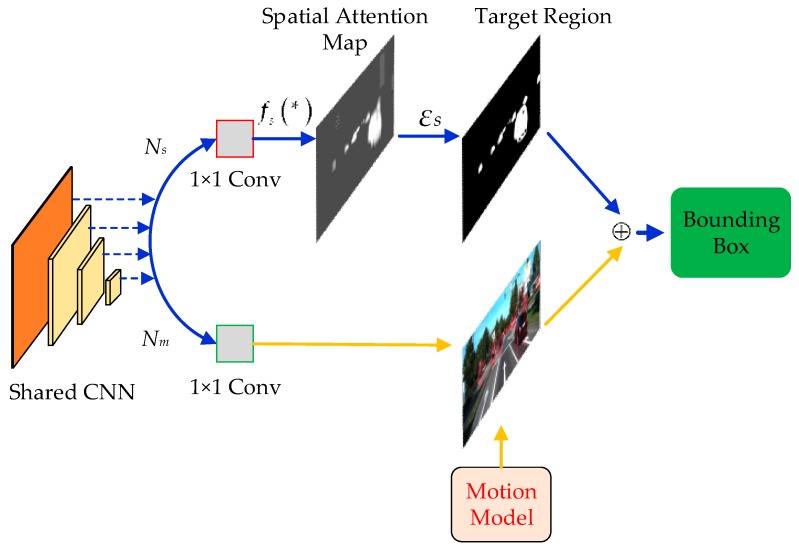
Offline Candidates Recommendation Module.

Detection is the cornerstone of multi-object tracking methods based on tracking-by-detection. With the great success of detectors based on deep convolutional neural networks, offline modules have been widely applied in detecting the stages of multi-object tracking. Among them, region proposal networks (RPNs) [30] are considered to be the most successful ROI proposal method, and are widely used in many detection applications [31,32,33,34,35]. In an RPN, anchors are defined as a set of sliding windows with fixed scales and aspect ratios [30]. In order to ensure a sufficiently high recall for proposals, a large number of anchors are used in such methods. Obviously, if this exhaustive strategy is adopted in two-step multi-object tracking, the process of estimating large numbers of candidates is extremely computation-expensive. The main reason for this is that most of the bounding box (or anchors) are placed in areas that do not contain targets. Inspired by Wang et al. [36], we adopt a spatial-attention map to recommend the candidates at the detection stage. As shown in Figure 3, our Offline Candidates Recommendation Module includes a shared features extraction CNN, a spatial-attention branch $N_{s}$ and a Motion Model and bounding box prediction branch $N_{m}$.

A smaller number of candidates containing all targets means a decrease in calculations of regression and classification. We present an effective and efficient scheme that leverages semantic features to guide the bounding box. As shown Figure 3, we use a spatial-attention branch $N_{s}$ and each feature map $F_{I}$ to generate a spatial-attention map $M_{s}$ of the target, which can be formulated as

(1) $$M_{s}=M\left(\cdot {|F}_{I}\right)=f_{s}\left(F_{I};\omega_{s}\right),M\left(\cdot {|F}_{I}\right)\in R^{w\times h},F_{I}\in R^{w\times h},$$

where, $\omega_{s}$ is the set of parameters in the spatial-attention branch $N_{s}$, and $f_{s}\left(*\right)$ is modeled as a 1×1 convolution with an element-wise sigmoid function. Each $M(i,j|F_{I})$ corresponds to the location with coordinate $\left(\left(i+\frac{1}{2}\right)s,\left(j+\frac{1}{2}\right)s\right)$, where s is the stride of the feature map. For each location aim-listed spatial-attention values, we adopt a global threshold $\varepsilon_{s}$ to determine whether the location belongs to a target, which can be formulated as

(2) $$\{\begin{array}{l} M\left(i,j{|F}_{I}\right)\geq \varepsilon_{s},\;\;\;Target;\\ M\left(i,j{|F}_{I}\right)<\varepsilon_{s},\;Background. \end{array}$$

**Figure 4:**
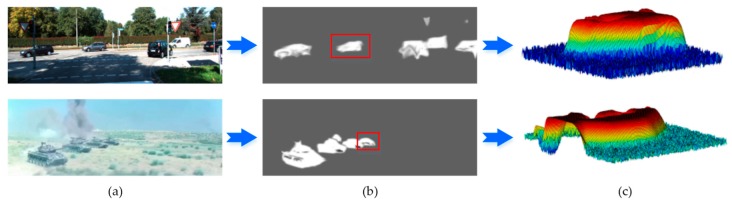
Example of the spatial-attention map and 3D spatial-attention value features of targets. (**a**) Input frames; (**b**) spatial-attention map; (**c**) 3D spatial-attention value features of targets.

According to the spatial-attention values of each position and the global threshold value $\varepsilon_{s}$, we determine the active regions where targets may possibly exist. This process can filter out about 80% of the regions while still maintaining the same recall. The determination of the bounding box shape and center location are introduced in the next chapter. Figure 4 shows an example of a spatial-attention map generated by the branch $N_{s}$ and 3D probability features of targets. In the spatial-attention map, the 3D probability features of targets are more prominent than background.

### 3.3. Motion Model

**Figure 5:**
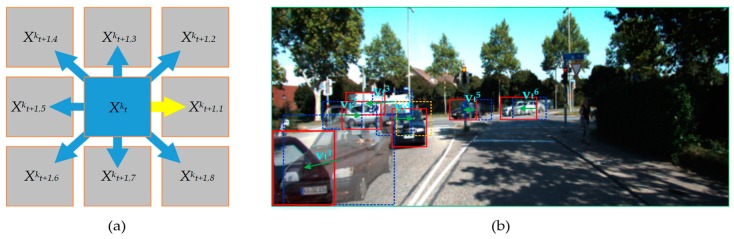
(**a**) Spatial positions of the relative predicted candidates at frame t+1; (**b**) example of the response of the same target’s candidate to the motion model at different frames.

A motion model analyzes the motion curve of the target in the history frames and predicts the position of the target in the current frame [37,38]. Most single-object trackers do not use a motion model [6,7,8,9]. However, the motion model has proven helpful in multi-object tracking, which can help locate targets and realize the correspondence of multi-target labels in different frames. In most MOT applications [29,39,40,41], a simple linear motion model is used to estimate the target state. Such motion models may cause a loss of tracking when the target turns quickly, suddenly stop or drives in reverse. In this work, we give full consideration to the possible motions of a target and propose a novel Motion Model to locate the candidates. In the spatial-attention branch $N_{s}$, we determine the possible areas of bounding boxes. The final position of candidates is determined with branch $N_{s}$ and the Motion Model. The estimated state of k_th candidate $C^{k}$ at t frame can be formulated as

(3) $$X_{t}^{k}=\left[x_{t}^{k},y_{t}^{k},w_{t}^{k},h_{t}^{k}\right],$$

where $x_{t}^{k}$ and $y_{t}^{k}$ represent the center location of the candidate, and $w_{t}^{k}$ and $h_{t}^{k}$ denote the width and height of candidate, respectively. In our Motion Model, the predicted state set $Q_{t+1}^{k}$ of $C^{k}$ at t+1 frame can be expressed as

(4) $$\begin{array}{l} Q_{t+1}^{k}={\left\{\tilde{X_{t+1,n}^{k}}\right\}}_{n=1}^{8}\\ \;\;\;\;\;=\left\{X_{t}^{k}+v_{t}^{k}\left({\left[\pm 1,0,0,0\right]}^{T}\;,{\left[0,\pm 1,0,0\right]}^{T},\;\frac{1}{2}{\left[\pm \sqrt{2},\pm \sqrt{2},0,0\right]}^{T},\;\frac{1}{2}{\left[\pm \sqrt{2},\mp \sqrt{2},0,0\right]}^{T}\right)\;\right\} \end{array}$$

where $\tilde{X_{t+1,n}^{k}}$ is the n_th predicted state of candidate $C^{k}$ at frame t+1, and $v_{t}^{k}$ represents the velocity of k_th candidate $C^{k}$ at frame t. Figure 5a shows the spatial positions of the relative predicted candidates at frame t+1. In order to cover the possible motion of the target, eight predicted states are used to formulated the candidates, which divide the direction of the space equally. Figure 4b shows an example of the response of the same target’s candidate to the motion model at different frames. In the figure, the green arrow represents the speed of the target, the blue dotted box represents the target bounding box at frame t, and the red box represents the target bounding box at frame *t* + 1. The response of target C^1^ is $X_{t+1,6}^{1}$ with a velocity $v_{t}^{1}$. In the same way, the response of target C^2^ is $X_{t+1,1}^{2}$ with a velocity $v_{t}^{2}$, and the response of target C^3^, C^5^ and C^6^ is $X_{t+1,5}^{k}$ (*k* = 3, 5, 6) with a velocity $v_{t}^{3}$, $v_{t}^{5}$ and $v_{t}^{6}$, respectively. For target $C^{4}$ with sudden turning, the orange dotted box represents the predicted position in the linear motion model and the red box represents the predicted position in our motion model. Obviously, our motion model had the better prediction ability, but a linear motion model cannot be applied to all situations.

In the spatial-attention branch $N_{s}$, a spatial-attention map $M_{s}$ is used to predict active regions where targets may possibly exist. We count the number of points that are greater than the threshold $\varepsilon_{s}$ in each predicted position and select the position with the most points that satisfies above condition as the response. The response process can be expressed as

(5) $$X_{t+1}^{k}=arg\underset{\tilde{X_{t+1,n}^{k}}\in M_{t+1}^{k}}{\max}\underset{M\left(i,j|X_{t+1,n}^{k}\right)\geq \varepsilon_{s}}{\sum }M\left(i,j|X_{t+1,n}^{k}\right),\;n=1,2,\ldots ,8.$$

Considering the occlusion, we take the direction of $v_{t}^{k}$ as the main direction. In this case, $X_{t+1}^{k}$ is formulated as

(6) $$X_{t+1}^{k}=X_{t}^{k}+\left[v_{t}^{k},0,\;0\right],$$

where $v_{t}^{k}=\frac{1}{T_{t+1}-T_{t}}(\left[x_{t+1}^{k},\;y_{t+1}^{k}{]}^{T}-{\left[x_{t}^{k},\;y_{t}^{k}\right]}^{T}\right)$ and $\left[x_{t}^{k},\;y_{t}^{k}\right]$ represent the center of candidate.

### 3.4. Online Candidates Estimation

Different from single-object tracking, the core research of multi-object tracking includes the occlusion of multiple targets, the insertion of new targets around boundaries and the disappearances of targets in the scene area. Occlusion is an important cue that needs to be considered during the online updating process. The appearance features of targets are polluted and cannot be used as online update samples when they are occluded by another target, building, fire or smoke. However, in the Motion Model and offline trained classifier, the covered position still scores highly. In this case, the corresponding tracker updates the appearance model with the corrupted samples and gradually drifts to the occluder or background.

The deep features are extracted from shared CNNs using ROI pooling, which ignores the occlusion. In order to address the occlusion, we use target-specific CNNs to estimate the candidates and classify the targets and background. The ROI-pooled feature representation of the k_th candidate $C^{k}$ is denoted as $\Phi_{roi}\left(X_{t}^{k}\right)\in R^{w\times h\times c}$. As in [3], a visibility map $X_{t}^{k}$ is output to encode the spatial visibility of the input samples, which can be expressed as

(7) $$V_{vis}\left(X_{t}^{k}\right)=f_{vis}\left(\Phi_{roi}\left(X_{t}^{k}\right);\omega_{vis}^{k}\right),\;V\left(X_{t}^{k}\right)\in R^{w\times h},$$

where $\omega_{vis}^{k}$ is the set of visibility parameters of the k_th target-specific CNN, and $f_{vis}\left(*\right)$ is modeled as both a convolutional layer (kernel size=3×7×32) and a fully connected layer (output size=w×h). We estimate the k_th candidate with an occlusion score $p_{t}^{k}$:

(8) $$p_{t}^{k}=f_{cls}\left(\Psi_{ref}\left(X_{t}^{k}\right);\;\omega_{cls}^{k}\right),\;p_{t}^{k}\in \left[0,1\right],$$

where $\omega_{cls}^{k}$ is the set of classification parameters of the k_th target-specific CNN, and $f_{cls}\left(*\right)$ is modeled as both a convolutional layer (kernel size=3×7×32) and a fully connected layer (output size=1). $\Psi \left(X_{t}^{k}\right)\in R^{w\times h\times c}$ denotes the refined features of the k_th candidate $C^{k}$, which is expressed as

(9) $$\Psi_{ref}\left(X_{t}^{k}\right)=\Phi_{roi}\left(X_{t}^{k}\right)\;\circ \;f_{con}\left(V_{vis}\left(X_{t}^{k}\right);\omega_{con}^{k}\right)\;\Psi_{ref}\left(X_{t}^{k}\right)\in R^{w\times h\times c}$$

where ∘ represents the channel-wise Hadamard product operation, $f_{con}\left(*\right)$ denotes a local connected layer with a spatial SoftMax layer, and $\omega_{con}^{k}$ is the set of connected parameters of the k_th target-specific CNN.

**Figure 6:**
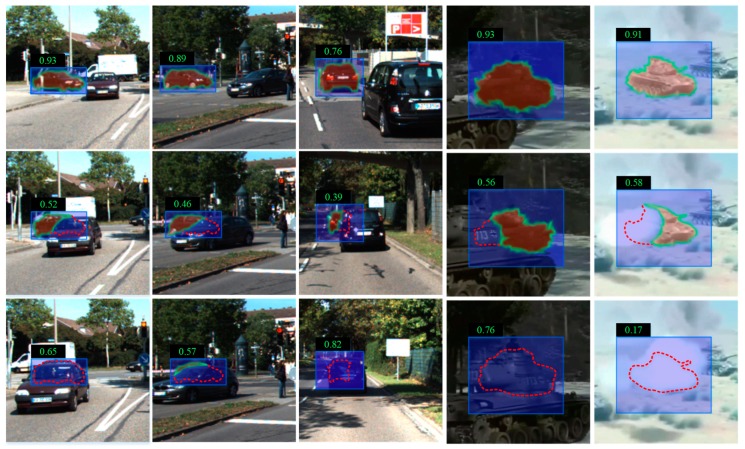
Examples of occlusion and generated visibility maps. The dotted lines surround the occluded targets. The blue bounding box is the predicted state set in the Motion Model. The numerical value is the classification score of candidates in the offline trained classifier.

Figure 6 shows examples of occlusion and generated visibility maps. The last column shows that the classification score is lower when the target is occluded by the background. However, when the target is occluded by a same-class target, the classification score is able to classify neither the tracked target nor the others. In the generated visibility maps, the degree of target occlusion is well evaluated, even if it is occluded by the same-class target. In this work, we use a threshold $p_{0}$ to estimate the degree of target occlusion. k_th candidates are taken as the tracking target without occlusion when $p_{t}^{k}\geq p_{0}$. On the contrary, k_th candidates are taken as an occluded target when $p_{t}^{k}<p_{0}$. $p_{0}$ is a classification threshold.

### 3.5. Temporal Attention Model

The polluted features of corrupted samples in bounding boxes would reduce the ability of estimation model to classify targets and backgrounds until the candidates cannot be evaluated. To address this conflict, a Temporal Attention Model is introduced in this work to balance the history and current frames in the online training process. As shown in Figure 2, the positive samples in history frames are saved in the Temporal Attention Model according to the scores of candidates in the estimation model. The history positive sample refers to a sample whose classification score is larger than the classification threshold ($p^{k}\geq p_{0}$), which reflects the original visual features of the target while the positive sample in current frame reflects the change of the visual features.

In this work, the Temporal Attention Model is used to update the Online Candidates Estimate Module and preserve historical positive samples. When the classification score of a candidate is larger than the classification threshold ($p^{k}\geq p_{0}$), the target is successfully tracked, and positive samples are used to update the online module—including the candidate of the same target in the current frame and the historical candidate in the Temporal Attention Model. The negative samples are all selected randomly from the current frame except for the candidate’s region. When the classification score of a candidate is lower than the classification threshold ($p^{k}<p_{0}$)—that is, the target is untracked—the positive samples used to update the online module all come from the Temporal Attention Model and the negative samples are all selected randomly from the current frame. For candidate $C^{k}$, the target-specific loss function in t frame can be expressed as

(10) $$L=L_{t}^{k-}+\lambda L_{t}^{k+}+\left(1-\lambda \right)L_{h}^{k+},$$

where λ is a temporal attention parameter to balance the current and history samples, which can be expressed as

(11) $$\lambda =\{\begin{array}{ll} 0 & p^{k}<p_{0}\\ 0.9 & p^{k}\geq p_{0} \end{array},$$

where $L_{t}^{k-}$ is the loss of negative samples in the current frame, $L_{t}^{k+}$ is the loss of positive samples in the current frame, and $L_{h}^{k+}$ is the loss of positive samples in the history frames. $L_{t}^{k-}$, $L_{t}^{k+}$, and $L_{h}^{k+}$ can be expressed respectively as

(12) $$L_{t}^{k-}=-\frac{1}{N_{t}^{k-}}\overset{N_{t}^{k-}}{\underset{i=1}{\sum }}log\left[1-f_{cls}\left(\Psi_{ref}\left(X_{t}^{k-}\right);\;\omega_{cls}^{k}\right)\right],$$

(13) $$L_{t}^{k+}=-\frac{1}{N_{t}^{k+}}\overset{N_{t}^{k+}}{\underset{i=1}{\sum }}logf_{cls}\left(\Psi_{ref}\left(X_{t}^{k+}\right);\;\omega_{cls}^{k}\right),$$

(14) $$L_{h}^{k+}=-\frac{1}{N_{h}^{k+}}\overset{N_{h}^{k+}}{\underset{i=1}{\sum }}logf_{cls}\left(\Psi_{ref}\left(X_{h}^{k+}\right);\;\omega_{cls}^{k}\right),$$

where $N_{t}^{k-}$, $N_{t}^{k+}$, and $N_{h}^{k+}$ are the number of negative and positive samples in the current frame and positive samples in history frames. In this work, we used a BP algorithm to update the weight parameters of each layer in the online estimation module.

### 3.6. Training of Module

#### 3.6.1. Training of the Offline Candidate Recommendation Module

In our Offline Candidate Recommendation Module, spatial-attention branch $N_{s}$ and a Motion Model are used to generate candidates. The offline module is optimized in an end-to-end fashion using a multi-task loss. In order to train the spatial-attention branch, a target location loss $L_{loc}$ is introduced based on Focal Loss [42]. At the initial stage of tracking, a bounding box prediction branch $N_{m}$ is used to generate a bounding box and solve the problem of generating the Motion Model. The conventional classification loss $L_{cls}$ and regression loss $L_{reg}$ are adopted to train the branch $N_{m}$. The two branches are jointly optimized with the following loss:

(15) $$L=L_{loc}+L_{cls}+L_{reg}$$

In the training of spatial-attention branch $N_{s}$, binary labeled maps are used as samples. In the binary labeled maps, 1 represents the valid location of a target center and 0 represents an invalid location. In this work, ground-truth bounding boxes are used to guide the generation of samples. Let ${\left(x_{g},y_{g},w_{g},h_{g}\right)}_{n}$ represent the mapped ground-truth bounding box in n_th feature map. The center region in the binary labeled map can be expressed as

(16) $$R_{center}=\left(x_{g},y_{g},0.1w_{g},0.1h_{g}\right)$$

The invalid region is the feature map excluding the mapped ground-truth bounding box, which can be expressed as

(17) $$R_{invalid}=F_{n}\setminus {\left(x_{g},y_{g},w_{g},h_{g}\right)}_{n}$$

where $F_{n}$ represents the n_th feature map.

#### 3.6.2. Training of the Online Candidate Estimation Module

In Online Candidate Estimation Module, we use target-specific CNNs to estimate the candidates and classify the target and background. The target-specific CNNs predict occlusion scores to estimate the candidates. At the initial stage of tracking, the parameters of the target-specific CNNs are random, and the networks have no estimation ability. In order to train the online module, we take the detection results of the Offline Candidate Recommendation Module as positive samples. Let ${\left(x_{d},y_{d},w_{d},h_{d}\right)}_{k}$ represent the k_th detection result and positive sample. Negative samples are constructed by positive samples and position offset. The k_th negative sample according to the positive sample can be formulated as

(18) $$Samples_{N}=\left\{{\left(x_{d}\pm \sigma_{1}w_{d},y_{d}\pm \sigma_{2}h_{d},w_{d},h_{d}\right)}_{k},{\left(x_{d}\pm \sigma_{1}w_{d},y_{d}\mp \sigma_{2}h_{d},w_{d},h_{d}\right)}_{k}\right\},$$

where $\sigma_{1}$ and $\sigma_{2}$ are randomly selected within the interval [0.7,0.9]. In order to achieve a robust performance, the online target-specific CNNs need sufficient samples to be trained. Denote the frame rate of the video as $N_{v}$ and use $N_{init}=0.2N_{v}$ to complete the training of the Online Candidate Estimation Module once the frames are sufficient. In actual training we use the first 20 frames to complete the training of the online module when the number of video frames is less than 100.

## 4. Experiment

### 4.1. Dataset and Implementation

In the multi-object tracking task, an initially unknown number of targets must be tracked as bounding boxes in a video. At present, multi-object tracking (MOT) datasets for general targets like pedestrians and vehicles have been published. The MOT Challenge datasets [43,44] show pedestrians from a variety of different viewpoints. The KITTI tracking dataset [45] features video from a vehicle-mounted camera and consists of 21 training sequences and 29 test sequences. However, such datasets do not contain particular armored targets or complex battlefield scenes (Figure 1). In this work, we built a dataset for armored targets, named the Armored Target Tracking Dataset (ATTD). The ATTD contained 80 (50 training, 30 test) video sequences in a complex battlefield scene, including various battlefield terrains (such as jungle, desert, grassland, and city) and complicated factors (such as armored clustering, muzzle fire and smoke, dust, and so on). All videos were captured by actual shooting and downloaded from the internet. All frames in the ATTD were normalized to a size of 1920 × 770 pixels. Armored target scales in the ATTD had wide range from 10 × 10 pixels to more than 700 × 700 pixels, with an emphasis on remote, small-armored targets. In this work, we use the KITTI training set and the ATTD to evaluate our MOT method for vehicles and armored targets.

In this work, pre-trained Resnet50 models [40] were used as the backbone network for the Offline Candidate Recommendation Module. The Offline Candidate Recommendation Module was trained with Adam, with a momentum of 0.9 and a weight decay of 0.0005, using a single NVIDIA GeForce GTX 2080ti GPU with 11 GB of memory. The Online Estimation Module was trained with the BP algorithm.

### 4.2. Evaluation Metrics

To evaluate the performance of our multi-object tracking method, we adopted the widely used CLEAR MOT metrics [46], including multiple-object tracking precision (MOTP) and multiple-object tracking accuracy (MOTA). MOTP represents the total error in estimated position for matched object-hypothesis pairs over all frames, averaged by the total number of matches made, which can be expressed as

(19) $$\mathrm{MOTP}=\frac{\sum_{k,t}d_{t}^{k}}{\sum_{t}N_{t}}$$

where $d_{t}^{k}$ is the distance between the k_th center of the ground-truth bounding box and its corresponding hypothesis in frame t. $N_{t}$ is the total number of matches made. MOTP reflects the ability of the multi-object tracker to estimate precise object positions, independent of its skill at recognizing object configurations, keeping consistent trajectories. MOTA can be seen as derived from three error ratios

(20) $$\mathrm{MOTA}=1-\left(\mathrm{FP}+\mathrm{FN}+\mathrm{IDS}\right)=1-\frac{\sum_{t}\left(m_{t}+fp_{t}+mme_{t}\right)}{\sum_{t}g_{t}}$$

where $m_{t}$, $fp_{t}$, and $mme_{t}$ are the number of false negatives (FN), false positives (FP) and identity switches (IDS), respectively. MOTA reflects all object configuration errors, including false positives, misses, and mismatches made by the multi-object tracker over all frames. Additionally, the percentage of mostly tracked targets (MT) and mostly lost targets (ML) are used as metrics in this work.

### 4.3. The Setting of Parameters

**Figure 7:**
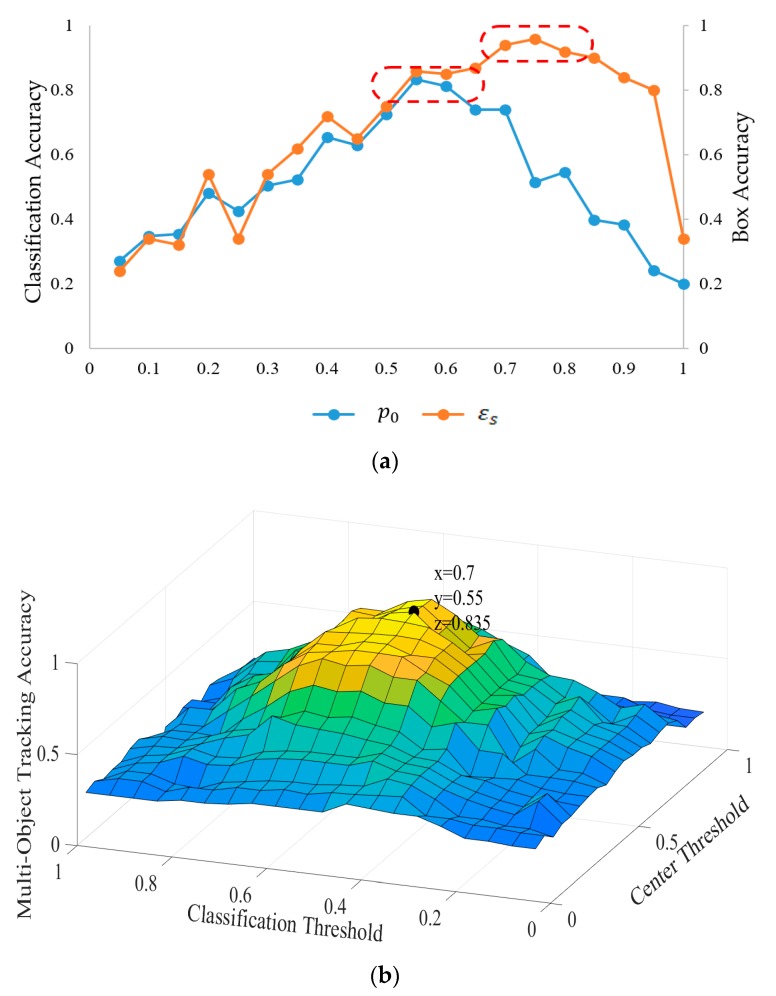
The selection of the global threshold $\varepsilon_{s}$ and classification threshold $p_{0}$. (**a**) The relationship between the global center threshold $\varepsilon_{s}$ and the predicted bounding box accuracy. The result of classification accuracy of armored targets determined by the classification threshold $p_{0}$. (**b**) The result of MOTA at different values of the global center threshold $\varepsilon_{s}$ and classification threshold $p_{0}$.

In our MOT algorithm, the birth/death of the trackers was determined by the global center threshold $\varepsilon_{s}$ and classification threshold $p_{0}$. The former determines whether a location belongs to a target and the latter determines the classification of armored targets and backgrounds and estimates the occlusion. In order to select appropriate parameters, we conducted an exhaustive experiment on the small training set, where several performance indicators are used for estimation. First, we randomly selected 1000 frames from the videos of the KITTI dataset. Half of the frames were used as training samples and the other half belonging to the same video were taken as test samples. Then, the prediction accuracy of the bounding box (Box Accuracy) of the Offline Candidates Recommendation Module was used to evaluate the threshold $\varepsilon_{s}$. The classification accuracy of positive samples (tracked targets) and negative samples (background, occlusion) in the Online Candidates Estimation Module was used to evaluate the threshold $p_{0}$. Meanwhile, we used MOTA as the joint performance indicator of $\varepsilon_{s}$ and $p_{0}$. The experimental results are shown in Figure 7. Figure 7a shows the variation of prediction accuracy of the bounding box (Box Accuracy) with the global threshold $\varepsilon_{s}$ and the result of classification accuracy of the target determined by the classification threshold $p_{0}$. When $\varepsilon_{s}$ lies in 0.65~0.9, the Offline Candidates Recommendation Module has a higher Box Accuracy. When $p_{0}$ lies in 0.5~0.7, the classification accuracy of the Online Candidates Estimation Module is higher. Therefore, in the above two intervals, we selected the appropriate threshold $\varepsilon_{s}$ and $p_{0}$ through the MOTA of the whole algorithm. Figure 7b shows the MOTA of our algorithm with different global threshold $\varepsilon_{s}$ and classification threshold $p_{0}$. The results demonstrate that the algorithm achieved the highest MOTA (83.5%) on the selected samples, when $\varepsilon_{s}=0.7\;\mathrm{and}\;p_{0}=0.65$. Hence, the next experiments were performed under the values listed above.

### 4.4. Analysis of Candidates Recommendation

In most MOT methods, an anchor-based RPN is the cornerstone of the object detection step. In order to ensure a sufficiently high recall for proposals, a large number of anchors are used in traditional detectors. Obviously, this scheme is extremely waste-computed. In this work, we adopted an alternative strategy to filter out most areas that were irrelevant to the objects of interest such as sky, grassland, desert and so on. In our MOT method, the ROI features of candidates were recommended by the Offline Candidates Recommendation Module and Motion Model. We used a spatial-attention branch $N_{s}$ and each feature map $F_{I}$ to generate a spatial-attention map $M_{s}$ of the target. A global threshold $\varepsilon_{s}$ was used to determine whether a location belonged to a target. The accurate positions of bounding boxes were further determined by assistance of the Motion Model. In order to demonstrate the ability of our Offline Candidates Recommendation Module and Motion Model, we studied the IoU distribution of proposals generated by three algorithms with different components. The details of each algorithm are described as follows:

M1: The shared features extraction CNN + “RPN + 9 anchors”;
M2: The shared features extraction CNN + $N_{s}$ + $\varepsilon_{s}$ + “RPN + 9 anchors”;
M3: The shared features extraction CNN + $N_{s}$ + $\varepsilon_{s}$ + Motion Model.

**Figure 8:**
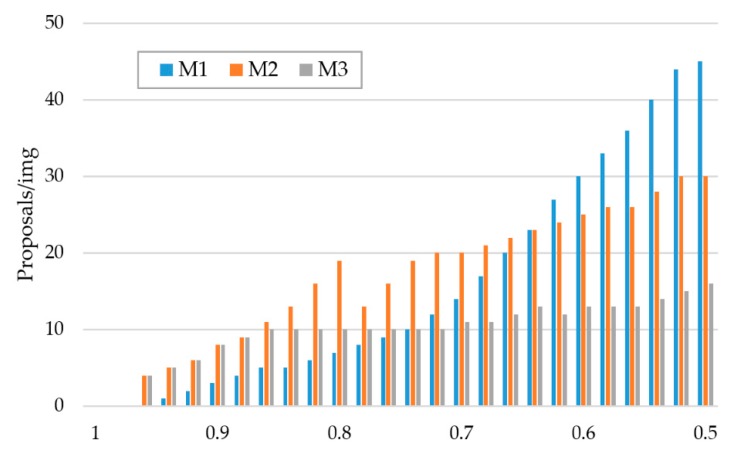
The IoU distribution of the M1, M2 and M3 proposals.

“RPN + 9 anchors” entails using three scales and three aspect ratios at each feature level. Figure 8 shows the IoU distribution of the three algorithms. The recommendation ability of the probability map is better than that of the RPN (M3≈M2>M1) when the IoU is set in a higher range (>0.8). Meanwhile, the number of proposals in M3 are significantly lower than the other two methods when the IoU is set in 0.5∼0.75. The reason for this is that using the spatial-attention map $M_{s}$ and global threshold $\varepsilon_{s}$ can result in filtering out most areas that are irrelevant to the object. In both datasets, the number of targets contained in each frame is generally 3∼5. Obviously, the excessive proposals of the RPN are redundant.

In order to further verify our candidate recommendation method, we compared MOTA, MOTP, MT, and ML for M1, M2 and M3 using the KITTI dataset, which is shown in Table 1. The comparison results of M1 and M2 show that, compared with a traditional RPN, our bounding box center prediction branch $N_{s}$ improved MOTA, MOTP, and MT by 10.85%, 1.13%, and 7.92%, respectively. The reason for this is that the branch $N_{s}$ filters out most areas that do not contain the target and reduces background interference. The comparison results of M3 and M2 show that our candidates recommendation method improved MOTA, MOTP, and MT by 13.13%, 14.13%, and 11.37%, respectively. This significant improvement clearly suggests that our Offline Candidates Recommendation Module and Motion Model are crucial for the detection step in multi-armored target tracking.

### 4.5. Analysis of Candidates Estimation

In our MOT method, the Online Candidates Estimation Module classifier recommended ROI features into targets and backgrounds and estimated the occlusion. The Temporal Attention Model saves history positive samples and updates the online module. The polluted features of corrupted samples in bounding boxes can reduce the ability of model estimates to classify targets and backgrounds until the candidates cannot be evaluated. In order to prevent the degradation of target-specific CNNs in the Online Candidates Estimation Module, we used the Temporal Attention Model to balance the current and history frames. In order to verify the validity of our Online Candidates Estimation Module and Temporal Attention Model, a comparison experiment involving three algorithms was conducted. The details of each algorithm are described as follows:

M3: The shared features extraction CNN + $N_{s}$ + $\varepsilon_{s}$ + Motion Model;
M4: M3 + Online Candidates Estimation Module;
M5: M3 + Online Candidates Estimation Module + Temporal Attention Model.

The comparison results are shown in Table 2. Compared with use of offline module only (M3), M4 improved MOTA by 15.87%, which demonstrates that our Online Candidates Estimation Module can effectively distinguish tracking targets from backgrounds. Compared with M4, M5 improved MOTA by 4.33%. The reason for this is that our Temporal Attention Model prevents the degradation of target-specific CNNs by balancing the history and current frames, which is illustrated in the significant reduction in ML by 13.11%. Meanwhile, MOTP and MT were also improved by 4.03% and 5.19% respectively.

**Figure 9:**
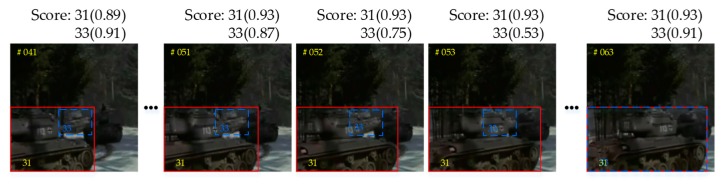
An example of drift caused by occlusion without the Temporal Attention Model.

Figure 9 shows an example of one target occluded by another when they are close to each other. The target-specific tracker gradually drifts to the occluder without the Temporal Attention Model. The classification score of target 33 decreases gradually, until it is occluded. The polluted features of target 33 gradually drift its bounding box to target 31.

### 4.6. Benchmark Evaluation Results

To demonstrate the effectiveness of our online multi-object tracking method, we compared our algorithm to several state-of-the-art approaches using both the KITTI and ATTD, including offline tracking methods like Siamese CNN [47], Convolutional Neural Networks and Temporally Constrained Metrics (CNNTCM) [48], Discrete-Continuous Energy Minimization (DCO-X) [49], and Learning Optimal Structured Support Vector Machine LP-SSVM [50], Online tracking methods like Near-Online Multi-Target Tracking (NOMT-HM) [51], Structural Constraint Event Aggregation (SCEA) [52], Spatial-Temporal Attention Mechanism (STAM) [3], Successive Shortest Path (SSP) [53], multi-modality Multi-Object Tracking (mmMOT) [54], and Multi-Object Tracking Beyond Pixels (MOTBeyondPixels) [55]. Some of these approaches could only be performed in the offline setting. We reimplemented these methods into our platform and used the average tracking time per frame as the tracking time.

Results using the KITTI dataset: The comparison results with the ATTD tracking testing set are summarized in Table 3. Our approach achieved the best MOTP, MT, and ML. Compared with the second best, we obtained 4.88% and 7.42% increases in MOTA and MT, respectively, and a 0.02% decrease in ML. Our method achieved the second best MOTP by 85.55%. Compared with online methods, the offline methods generally had a longer tracking time. Further, in most cases, there were not enough samples to train the offline model in MOT tracking. The higher MOTA and MT indicate that our Offline Candidates Recommendation Module filtered out most areas that did not contain the target, reduced background interference, and was crucial for the detection step. The best MOTP indicates that the Motion Model could accurately predict the trajectory of motion. The decrease in ML indicates that our approach had fewer false negatives, which should be largely attributed to our Temporal Attention Model preventing the degradation of target-specific CNNs by balancing the history and current frames. The overall running time indicates that our approach met the actual requirements.

**Figure 10:**
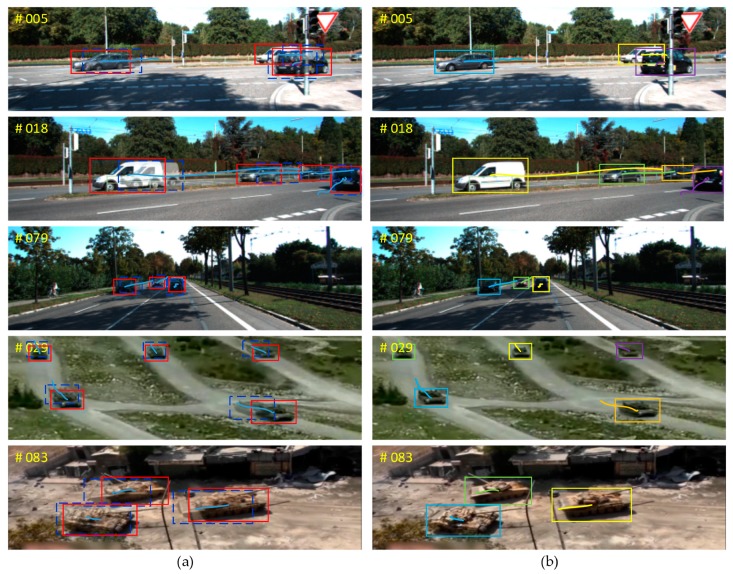
Experiment results using both the KITTI dataset and the ATTD. (**a**) Detection results with our Offline Candidates Recommendation Module. The previous position is denoted with a blue rectangle and the current with a red. (**b**) Tracking results using our MOT method and target trajectories.

Results using the ATTD: The comparison results using the ATTD tracking testing set are summarized in Table 4. Our approach achieved the best MOTP, MT and ML. Compared with the second best, we obtained 3.45%, 4.04%, and 2.71% increases in MOTA, MOTA, and MOTP, respectively, and a 3.98% decrease in ML. Figure 10 shows some qualitative results of our MOT method using both KITTI and ATTD, where we merged two sequential frames to make their difference apparent. The object trajectories are also shown in the figure.

## 5. Conclusions

The tracking of multiple objects can be complicated by occlusion, insertion among targets, complex backgrounds, and real-time requirements. Moreover, there is no ready-made multi-object tracking (MOT) dataset for armored targets. In this work, we proposed an online multi-object tracking method and a special MOT dataset for armored targets, named the Armored Target Tracking Dataset (ATTD). Combining the exhaustive strategy of traditional RPN with new target insertion, we used an Offline Candidates Recommendation Module to recommend candidates in the detection stage. The offline module adopted a spatial-attention branch $N_{s}$ to filter out most areas that were irrelevant to the objects of interest. A novel Motion Model was proposed to assist in locating the candidates and provide full consideration to the possible motion of the target. Significant improvements in several comparison experiments clearly suggests that our Offline Candidates Recommendation Module and Motion Model were crucial to the detection step in multi-object tracking. In order to address the occlusion among targets, we used target-specific CNNs to estimate the candidates in the Online Candidates Recommendation Module, which estimates target occlusion and classification. In order to prevent the polluted features of corrupted samples from reducing the ability of the estimation model, a Temporal Attention Model was introduced to balance the history and current frames in the online training process. Our Online Candidates Estimation Module could effectively distinguish tracking targets from the background. The Temporal Attention Model prevented the degradation of target-specific CNNs by balancing the history and current frames. Experimental results show that our method achieved outstanding increases in MOTA, MOTP, and MT, and decreased ML. The overall running time indicates that our approach was able to meet the requirements of the experiment. In the future, we will test this method with other MOT datasets and consider strengthening the detection by merging visible and infrared images.

## Figures and Tables

**Table 1:** Analysis of the Offline Candidates Recommendation and Motion Model using the KITTI dataset.

Method	MOTA	MOTP	MT	ML
M1(Shared CNN + RPN + 9 anchors)	45.47%	65.30%	27.54%	19.35%
M2(M1 + $N_{s}$ + $\varepsilon_{s}$)	56.32%	66.43%	35.46%	18.36%
M3(Shared CNN + $N_{s}$ + $\varepsilon_{s}$ + Motion Model)	69.45%	80.56%	46.83%	17.25%

**Table 2:** Analysis of the Offline Candidates Recommendation and Motion Model using the KITTI dataset.

Method	MOTA	MOTP	MT	ML
M3(Shared CNN + $N_{s}$ + $\varepsilon_{s}$ + Motion Model)	69.45%	80.56%	46.83%	17.25%
M4(M3 + Online Candidates Estimation)	85.32%	81.52%	75.46%	15.36%
M5(M4 + Temporal Attention Model)	89.65%	85.55%	80.65%	2.25%

**Table 3:** Comparison with state-of-the-art methods using the KITTI tracking testing set.

Method	Mode	MOTA	MOTP	MT	ML	Tracking Time (s)
Siamese CNN	offline	46.31%	71.20%	15.52%	27.30%	0.81
CNNTCM	offline	49.50%	71.80%	19.75%	22.64%	0.73
LP-SSVM	offline	75.65%	77.80%	42.54%	10.25%	0.95
mmMOT	offline	84.77%	85.21%	73.23%	2.77%	0.16
NOMT-HM	online	69.12%	78.52%	38.51%	15.28%	0.09
STAM	online	77.20%	74.90%	29.65%	18.57%	0.25
SSP	online	68.00%	79.52%	42.05%	10.64%	0.61
MOTBeyondPixels	online	84.24%	85.73%	73.23%	2.77%	0.30
Ours	online	89.65%	85.55%	80.65%	2.25%	0.16

**Table 4:** Comparison with state-of-the-art methods on the ATTD tracking testing set.

Method	Mode	MOTA	MOTP	MT	ML	Tracking Time (s)
SiameseCNN	offline	46.31%	71.20%	15.52%	27.30%	0.81
CNNTCM	offline	49.50%	71.80%	19.75%	22.64%	0.73
DCO-X	offline	65.12%	73.85%	31.52%	14.25%	0.95
LP-SSVM	offline	75.65%	77.80%	42.54%	10.25%	0.16
NOMT-HM	online	69.12%	78.52%	38.51%	15.28%	0.09
SCEA	online	34.35%	71.10%	47.35%	37.50%	0.18
STAM	online	77.20%	74.90%	29.65%	18.57%	0.25
SSP	online	68.00%	79.52%	42.05%	10.64%	0.61
Ours	online	80.65%	83.55%	49.52%	6.27%	0.26
